# Retraction: Perthamide C Inhibits eNOS and iNOS Expression and Has Immunomodulating Activity In Vivo

**DOI:** 10.1371/journal.pone.0222175

**Published:** 2019-08-30

**Authors:** The PLOS ONE Editors

Following publication of this article [1] the following concerns were raised:

- Figure 3A eNOS panel lanes 1 and 2 appear similar within the bands and the surrounding background when adjusted for brightness.
- Figure 3A β-actin panel contains a vertical discontinuity between perthamide C treated 72h and 96h lanes.
- Figure 3 and Figure 4 β-actin control panels appear similar.

The authors provided supporting image data underlying Figures 3A (eNOS), 3B (iNOS), 3B/4A (β-actin), and 4B (COX-2) as well as replication data for the eNOS and COX-2 experiments. Supporting image data for Figure 2A (eNOS, β-actin) and individual level data underlying all graphs in the article are available from the authors.

The authors explained that the same β-actin control panels were used in Figures 3A and 4B, and in Figures 3B and 4A, as the same gels were used to examine expression of COX and NOS proteins. Blots shown in Figures 3A and 4B are derived from the same gel used to examine eNOS and COX-2 expression, and blots shown in Figures 3B and 4A are derived from the same gel used to examine iNOS and COX-1 expression.

The data provided did not resolve the first two concerns listed above about Figure 3A. In light of these unresolved concerns, the *PLOS ONE* Editors retract this article.

GC notified the journal that the authors disagree with the retraction. MB, VV, EP, AI confirmed their disagreement, the other listed authors did not respond directly to the journal’s notification or could not be reached.

## References

[1] Bucci M, Cantalupo A, Vellecco V, Panza E, Monti MC, Zampella A, et al (2013) Perthamide C Inhibits eNOS and iNOS Expression and Has Immunomodulating Activity In Vivo. PLoS ONE 8(3): e57801 10.1371/journal.pone.0057801 23554869 PMC3595234

